# Persistant genital arousal disorder and venlafaxine: A case report

**DOI:** 10.1192/j.eurpsy.2021.1464

**Published:** 2021-08-13

**Authors:** M. Marguilho, M. Gonçalves, I. Pereira, G. Marinho, A. Nobre

**Affiliations:** Clínica 5, Centro Hospitalar Psiquiátrico de Lisboa, Lisboa, Portugal

**Keywords:** persistent, venlafaxine, genital, sexual

## Abstract

**Introduction:**

In this presentation we describe the case of a woman referred to the Sexology Department after having developed symptoms of Persistent Genital Arousal (PGAD) for the last 5 years, during treatment for Depression with Venlafaxine. PGAD is a clinical entity first described in 2001 by Leiblum and Nathan. Despite having received more attention in the last few years, its etiology remains unclear, with numerous causal factors of different natures being suggested.

**Objectives:**

We aim to describe this clinical case of PGAD and to discuss the possible etiological factors involved as well as to make a brief revision of the literature on this topic.

**Methods:**

We conducted a detailed interview, focused on the nature of the complaints, psychological history, medications, diet and neurologic disorders and performed a thorough clinical examination. We also searched for relevant articles in medical databases such as PubMed and Google Scholar.

**Results:**

A 52 year-old woman previously treated for Depression with Venlafaxine complains of involuntary sensations of genital arousal, with perceived vasocongestion, tingling and pulsatlity during her journey to work in public transportations. The symptoms subsided only after getting home 8-10 hours later and reaching orgasm by masturbating. She stopped Venlafaxine in 2015, but these symptoms persisted. Some authors suggest a link between SSRIs/SNRIs and PGAD.

**Conclusions:**

PGAD is a relatively recent addition to our diagnostic catalog with increasingly more cases being reported in the last few years. It is likely that the condition, however, has no discrete etiology and that a customized approach will be necessary to successfully treat most patients.

